# Hospital-acquired and ventilator-associated pneumonia caused by multidrug-resistant Gram-negative pathogens: Understanding epidemiology, resistance patterns, and implications with COVID-19

**DOI:** 10.12688/f1000research.129080.2

**Published:** 2024-03-25

**Authors:** Dalal Hammoudi Halat, Carole Ayoub Moubareck

**Affiliations:** 1 Academic Quality Department, QU Health, Qatar University, Doha, Qatar; 2 College of Natural and Health Sciences, Zayed University, Dubai, United Arab Emirates

**Keywords:** hospital-acquired pneumonia; ventilator-associated pneumonia; antimicrobial resistance; Gram-negative multi-drug resistant pathogens.

## Abstract

The ongoing spread of antimicrobial resistance has complicated the treatment of bacterial hospital-acquired pneumonia (HAP) and ventilator-associated pneumonia (VAP). Gram-negative pathogens, especially those with multidrug-resistant profiles, including *Escherichia coli*, *Klebsiella pneumoniae*, *Enterobacter* spp., *Pseudomonas aeruginosa*, and *Acinetobacter* spp., are important culprits in this type of infections. Understanding the determinants of resistance in pathogens causing pneumonia is ultimately stressing, especially in the shadows of the COVID-19 pandemic, when bacterial lung infections are considered a top priority that has become urgent to revise. Globally, the increasing prevalence of these pathogens in respiratory samples represents a significant infection challenge, with major limitations of treatment options and poor clinical outcomes. This review will focus on the epidemiology of HAP and VAP and will present the roles and the antimicrobial resistance patterns of implicated multidrug-resistant (MDR) Gram-negative pathogens like carbapenem-resistant *Acinetobacter baumannii* (CRAB), carbapenem-resistant *Pseudomonas* *aeruginosa* (CRPA), carbapenem-resistant *Enterobacterales* (CRE), as well as colistin-resistant Gram-negative pathogens and extended-spectrum β-lactamase (ESBL)-producing *Enterobacterales.* While emerging from the COVID-19 pandemic, perspectives and conclusions are drawn from findings of HAP and VAP caused by MDR Gram-negative bacteria in patients with COVID-19.

## Introduction

Originating from the ancient Greek “pneumon”, or lung, pneumonia is defined as inflammation of the parenchyma of either one or both lungs, which is usually, but not always, caused by infection, and remains a foremost cause of hospitalization among both adults and children, ^1^ with a hospitalization rate close to 400 per 100 000 population in the United states ^2^ and over seven million hospitalizations per year worldwide. ^3^ Pneumonia must be considered in patients with acute onset of fever, chills, cough, dyspnea, fatigue, purulent sputum, anorexia, and pleuritic chest pain. ^4^ Pneumonia represents the eighth most expensive condition for hospitalization, with an estimated total cost exceeding USD 9.5 billion per year in US hospitals. ^5^

Although a causative pathogen may stay unrecognized, bacteria, viruses, fungi, and parasites may be implicated in pneumonia. ^6^ While viruses like influenza A and B, coronaviruses, rhinoviruses, respiratory syncytial viruses, parainfluenza viruses, and others, are vey frequent pathogens in pneumonia, ^6^^–^^8^ bacterial pneumonia continues to be one of the most serious public health problems due to its medical and economic burden. ^9^

According to the American Thoracic Society, ^10^ pneumonia can be classified into community-acquired pneumonia (CAP), hospital-acquired pneumonia (HAP), and ventilator associated pneumonia (VAP). While any pneumonia acquired outside the hospital or in community settings may be considered as CAP, HAP is defined as a pneumonia occurring 48 hours or more after hospital admission, and which was not incubating at the time of admission. On the other hand, VAP refers to pneumonia occurring more than 48–72 hours after endotracheal intubation. ^10^^,^^11^ As of 2016, the term “healthcare-associated pneumonia” (HCAP) is no longer recognized in medical literature. This category of pneumonia was referred to cases acquired in healthcare facilities including nursing homes, hemodialysis centers, and outpatient clinics, or during hospitalization within the past 90 days. ^11^ HCAP was a condition used to identify patients at risk of pneumonia caused by multidrug-resistant (MDR) pathogens depending upon specific risk factors and illness severity. However, this categorization appeared to be excessively sensitive and may have led to increased, inappropriate, broad-spectrum antibiotic use. Although patients having recent contact with healthcare facilities were at a higher risk for infection with such MDR pathogens, this risk remained small for most patients and the overall incidence of these pathogens was low, since it did not exceed 7% in high quality studies, and did not directly relate to patient mortality. ^12^^–^^14^ Accordingly, purposeful removal of the category of HCAP was done in 2016, and it was not classified as a discrete type of pneumonia in the 2017 guidelines on the management of HAP and VAP from Europe and Latin America. ^15^

Despite the availability of guidelines for management of HAP and VAP, ^16^ and the growing trend in understanding these infections, successful treatment remains complex to achieve, ^17^ and the incidence does not seem to be decreasing. ^18^ In this review, both the epidemiological and microbiological properties of HAP and VAP shall be highlighted, with focus on implicated Gram-negative MDR pathogens and their resistance patterns. Finally, and in the light of COVID-19, the importance of these pathogens in HAP and VAP shall be discussed according to relevant literature reporting them in the wake of the global pandemic. Literature for this narrative review was collected by a thorough search for published articles about MDR pathogens in HAP and VAP on PubMed and Google Scholar, to allow consideration of a broad collection of available evidence, and considering different countries, ages, and hospital settings. Also, articles published during COVID-19 were specifically considered to describe the status of these pathogens in VAP and HAP in light of the changes brought about by the pandemic.

## Risk factors and epidemiology of HAP and VAP

HAP and VAP represent some of the most common and serious infections occurring in hospitalized patients, ^19^ and remain important causes of morbidity and mortality despite advances in antimicrobial therapy, numerous supportive care modalities, and the use of a widerange of preventive measures. ^11^^,^^18^ Although the Centers for Disease Control and Prevention (CDC) estimate that over two-thirds of HAP in the United States occur in non-ventilated patients, there is a major gap in research on HAP, as most hospitals routinely track VAP, and most of the knowledge we have about nosocomial pneumonia including incidence, risk factors, mortality and prevention come from ventilated patients. ^20^ The most significant risk factors for HAP are old age ^21^ and comorbidities including coronary heart disease, diabetes, chronic lung disease, chronic renal failure, and thyroid disorders. ^22^^,^^23^ Mechanical ventilation, ^24^ especially if prolonged for more than two weeks, ^21^ reintubation or tracheostomy, ^25^ major chest or abdominal surgery, ^26^ as well as previous antibiotic exposure, especially to broad-spectrum antibiotics, ^27^ are possible risk factors for VAP. Of note, some studies have reported an increased incidence of HAP when the gastric pH is increased with the use of medications including H _2_ receptor blockers, antacids, or proton pump inhibitors. ^28^^,^^29^

HAP remains a highly prevalent and morbid hospital-acquired infection, second only to nosocomial bloodstream infections, ^30^ and its incidence ranges from 5-20 cases per 1000 hospital admissions, with highest rates observed among immunocompromised, surgical and elderly patients. ^31^ It is reported to affect nearly 0.5 to 1.7% of all hospitalized patients, and to be the leading cause of mortality among all hospital-acquired infections. ^9^ HAP frequently causes prolonged hospital stay, increased antimicrobial usage, and additional cost of treatment. ^32^

VAP is the most prevalent nosocomial infection in the intensive care unit (ICU), where it constitutes about 25% of ICU infections. ^33^ About 10% of patients on mechanical ventilation may develop VAP. ^34^ Moreover, according to results of meta-analysis, the attributable mortality rate in VAP was higher for surgical patients and those with severe illness at the time of admission. ^35^ Patients with VAP usually have a longer hospital course and excess mortality, and invite higher healthcare costs than similarly ill patients who do not have VAP. ^16^

Among ICU patients, both HAP and VAP are associated with high morbidity and mortality rates, since these patients are already weak and critically ill. The estimated all-cause mortality in such patients is between 25-50%. Globally, HAP and VAP are considered the leading causes of death due to hospital-acquired infection, with an estimated global mortality of 20–30% due to HAP, and 20–50% due to VAP. ^36^ In a comparative analysis of longitudinal prospective studies, and in the ICU setting, HAP and VAP were responsible for 82% and a 38% rise in the risk of 30-day mortality respectively. ^37^ With the current standards of therapy, the clinical success rates for patients admitted to the ICU with HAP or VAP are often below 60%, and this may be explained by challenges of antibiotic therapy in critically ill patients, complexity of identifying microbial etiologies, relatively low penetration of most antibiotics into the lungs, as well as frequency of difficult-to-treat or highly resistant pathogens in that setting. ^38^

## Microbiology of HAP and VAP: The role of MDR Gram-negative pathogens

Concise knowledge of the microbial etiologies of HAP and VAP allows better identification of patients at high risk of infection caused by problematic pathogens, such as MDR Gram-negative and extended spectrum beta-lactamase (ESBL)-producing *Enterobacterales*, in addition to carbapenemase-producing Gram-negative bacteria. This should guide better selection of antibiotics and assessment of treatment protocols. ^39^ A persistent clinical dilemma regarding the causes of HAP and VAP resides in that detection of a microorganism from a respiratory tract sample does not necessarily indicate it as the causative agent of pneumonia. ^40^ HAP and VAP may be caused by a wide variety of pathogens and can be polymicrobial.

Evidence indicates that *Streptococcus pneumoniae*, *Staphylococcus aureus*, *Klebsiella pneumoniae*, *Haemophilus infuenzae*, *Pseudomonas aeruginosa*, *Moraxella catarrhalis*, and *Escherichia coli* were the most identified causes of typical pneumonia, while atypical pneumonia is mostly attributed to pathogens like *Legionella pneumophila*, *Chlamydia pneumoniae*, and *Mycoplasma pneumoniae.* Despite the fact that *S. pneumoniae* is the most important cause of CAP globally, Gram-negative bacteria are commonly related to HAP and VAP. ^9^ In general, the implicated pathogens in HAP and VAP are *S. aureus* (especially methicillin-resistant *S. aureus* (MRSA) strains), *Pseudomonas* species (especially *P. aeruginosa*), *Acinetobacter* species, *E. coli*, in addition to *Klebsiella* species (including extended-spectrum β-lactamase (ESBL)-producing and the extensively drug-resistant (XDR) *Enterobacterales*). These pathogens account for nearly 80% of all episodes. ^41^^,^^42^

HAP etiology is thought to have shifted towards Gram-negative pathogens, ^9^ and this may be perpetuated by overuse of existing antimicrobial agents, which has led to the development of adaptive resistance mechanisms by bacteria; lack of good antimicrobial stewardship resulting in increased resistance; and lack of adequate infection control practices. ^43^^–^^45^

The involvement of Gram-negative bacteria in HAP and VAP differs across studies in different world regions. In a systematic review and meta-analysis conducted on Asian countries, and among a sum of 14295 organisms identified in VAP, the most predominant culprit was *Acinetobacter baumannii* (26%), followed by *P. aeruginosa* (22%), *K. pneumoniae* (14%), and *S. aureus* (14%). ^46^ Similarly, in the Southeast Asian region, a part of the world with limited health resources and underestimation of infectious diseases, a comparison of causative agents of VAP among 24 different studies showed that *Acinetobacter* spp., followed by *P. aeruginosa*, and then *K. pneumoniae* were the commonest Gram-negative organisms implicated in the disease. ^47^

In European ICUs, and according to a prospective, multicenter, observational study on HAP and VAP, the most common isolates identified were *S. aureus*, with a prevalence of 16% for methicillin-sensitive and 16% for methicillin-resistant, *P. aeruginosa* (23%), and *A. baumannii* (19%). ^48^ In a large study enrolling patients with VAP from 27 ICUs in nine European countries, the dominant isolates were *S. aureus* in Spain, France, Belgium and Ireland, *P. aeruginosa* in Italy and Portugal, *Acinetobacter* in Greece and Turkey, and *E. coli* in Germany. ^49^ Other reports document the high prevalence of *Enterobacterales* in nosocomial pneumonia, like a report from Poland ^50^ citing 42.0 % of *Enterobacterales*, 37% of *A. baumannii*, 16% of *P. aeruginosa*, and 5% of *Stenotrophomonas maltophilia.* In 2017, and in an investigation from Serbia on HAP and VAP causes, Gram-negative agents were mostly isolated; the most common pathogens were *Acinetobacter* spp. and *P. aeruginosa,* accounting for over 60% of isolates. ^51^ Similarly, in a 10-year surveillance study in a tertiary medical center in Lebanon on VAP causes, Gram-negative organisms were predominant among isolated pathogens (95%), with A. *baumannii* being the leading culprit (33%), followed by *P. aeruginosa* (17%) and *E. coli* (12%). ^52^ Similar results were reported in Saudi Arabia in a 6-year analysis by El-Saed and Colleagues. ^53^ In a study from the United Arab Emirates involving a 20-bedded, mixed, medical and surgical ICU, *K. pneumoniae* was the most prevalent organism (21%) in VAP, followed by *S. aureus* (16%), and *P. aeruginosa* (16%). ^54^

In the United States, and in an antimicrobial surveillance program in 2010 intended to establish the pathogens most likely to cause HAP or VAP, a consistent group of 6 organisms ( *S. aureus* [28.0%], *P. aeruginosa* [21.8%], *Klebsiella* species [9.8%], *E. coli* [6.9%], *Acinetobacter* spp. [6.8%], and *Enterobacter* spp. [6.3%]) caused approximately 80% of HAP or VAP. Lower prevalence of *Serratia* species, *S. maltophilia*, and community-acquired pathogens, such as pneumococci and *Haemophilus influenzae* were reported. ^55^ In 2016, a report by the Centers of Disease Control and Prevention (CDC) ^41^ indicated a similar ranking in VAP for *S. aureus*, *P. aeruginosa*, and *Klebsiella* spp., followed by *Enterobacter* spp. and *E. coli.* The incidence was different in an investigation carried in the ICU on patients with HAP or VAP, with *S. maltophilia* being most prevalent (34%), followed by *P. aeruginosa* (40%), *A. baumannii* (32%), and *S. aureus* (28%). ^56^ As such, the etiological diagnosis of bacterial causes of HAP and VAP shows variable distribution of frequent pathogens, underlining the necessity for incessant, vigilant monitoring of these data across the continuum of HAP and VAP in different areas worldwide.

## Resistance patterns of the important MDR Gram-negative bacteria implicated in HAP and VAP

**Figure 1.**
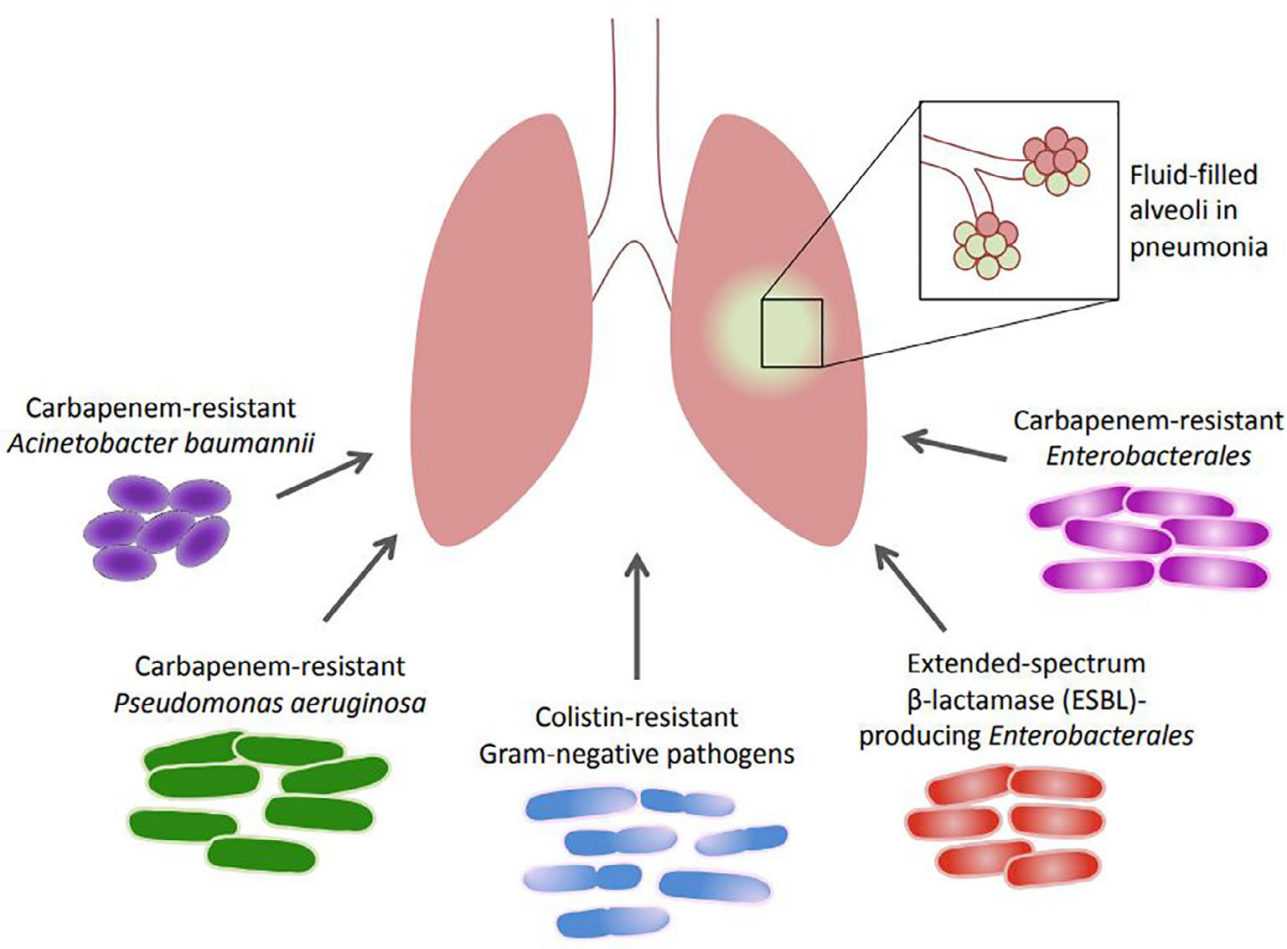
Incriminated Gram-negative bacteria in HAP and VAP.

The roles and the antimicrobial resistance patterns of extended-spectrum β-lactamase (ESBL)-producing *Enterobacterales*, carbapenem-resistant *Enterobacterales* (CRE), carbapenem-resistant *A. baumannii* (CRAB), carbapenem-resistant *P. aeruginosa* (CRPA), and colistin-resistant Gram-negative pathogens, pathogens incriminated in HAP and VAP, are summarized, and presented in Figure 1. This is especially important in light of the World Health Organization (WHO) classification of some of these pathogens as critical priority bacteria and urgent threats that should be addressed appropriately. ^57^

## Extended-spectrum β-lactamase (ESBL)-producing *Enterobacterales*

HAP and VAP due to ESBL-producing *Enterobacterales* represent a growing problem. Indeed, ESBL producers are endemic in many countries, and 5 to 25% of ICU patients are carriers of these pathogens on admission. HAP and VAP caused by ESBL-producers are associated with a higher mortality than HAP and VAP due to susceptible *Enterobacterales* because the resistance profile decreases the adequacy rate of empiric therapy. ^58^ The spread of pathogens harboring ESBLs with concomitant resistance to carbapenems further complicates treatment outcomes. ^59^ On molecular level, genes encoding carbapenem resistance, colistin resistance, and ESBLs are carried on highly mobile genetic elements, making them capable of easy transfer horizontally among bacteria with resulting additional dissemination and increase in resistance rates. ^60^

ESBL-producing *Enterobacterales* have been repeatedly described in HAP and VAP. In one study from Croatia, 4% of *K. pneumoniae* isolates and 2% of *E. cloacae* isolates were producers of ESBL, with group 1 CTX-M β-lactamases detected in patients with VAP. ^61^ In another report from France, ESBL-producing Enterobacterales accounted for 27% of VAP cases in a monocenter retrospective study among mechanically ventilated patients in the ICU. ^62^ Similar findings were reported in another study from Italy, were both carbapenemase-producing and ESBL-producing *K. pneumonia* were identified in patients with VAP in the ICU. ^63^

## Carbapenem-resistant *Enterobacterales* (CRE)

The *Enterobacterales* are frequently isolated in clinical cultures and are typical inhabitants of the digestive system in both humans and animals. Globally, the threat posed by CRE to human health is on the rise, imposing an urgent antimicrobial resistance threat. When compared to strains that are carbapenem-susceptible, CRE frequently have numerous resistance genes that restrict treatment options, entail longer treatment durations, impose higher costs, and require therapies with higher toxicities. ^64^ In fact, *Enterobacterales* can become resistant to carbapenems by three possible mechanisms: efflux pump overactivity, outer membrane porin loss or mutation, and carbapenemase production, which remains the major resistance mechanism. ^65^ Efflux pumps belonging to the resistance-nodulation-division (RND) are reported to contribute to carbapenem resistance, such as the common AcrAB-TolC RND system. ^66^ Furthermore, reduced carbapenem susceptibility in *Enterobacterales* may result from reduced expression of outer membrane porins such as OmpF and OmpC, which mediate drug permeability. ^67^ However, carbapenemases remain the key determinants of resistance in CRE and they belong to different Ambler classes: Class A like KPC, Class B like NDM, IMP and VIM, and class D like OXA enzymes. ^68^ Noteworthy, CRE with high efflux activity or permeability defects may express any of these mechanisms paired to production of other β-lactamases such as AmpC cephalosporinases or extended-spectrum β-lactamases (ESBLs), indicating the heterogeneity of mechanisms driving the development of carbapenem resistance. ^69^

In pneumonia, antibiotic resistance profiles of *Enterobacterales* have gradually changed, indicating that close monitoring of those pathogens is fundamental for preventing further rise of resistance, development of treatment guidelines, and improving clinical therapy. For example, in China, an analysis of CRE in HAP over one decade showed an increase in the prevalence of these organisms from 0.8% to 11.6%. ^70^ In 2021, a prospective cohort study involving 5 tertiary referral centers in Korea detected 4% prevalence of CRE in HAP and 1% in VAP. ^71^ In another investigation, both carbapenem-resistant *K. pneumoniae* encoding OXA-48, and carbapenem-resistant *Enterobacter cloacae* encoding VIM-1, were identified in VAP, among a diverse group of β-lactamases. ^61^

## Carbapenem-resistant *A. baumannii* (CRAB)

*A. baumannii* is an organism with extensive resistance to antimicrobial agents and with a profile perfectly compliant to healthcare settings, where it grows as cause of HAP and VAP. ^72^ Carbapenem resistance in *A. baumannii* is convened by several mechanisms acting in concert, including decrease in its outer membrane permeability, overactivity of efflux pumps, and increased production of cephalosporinases belonging to the AmpC group. However, the most predominant mechanism of carbapenem resistance in *A. baumannii* remains the production of carbapenemases of Ambler classes B and D. ^68^ Moreover, there are also reports of specific Ambler class A carbapenemases among *A. baumannii* strains. ^73^

The earliest case report of CRAB in nosocomial pneumonia was described in a mechanically ventilated patient in the ICU of a Spanish hospital in 1998. ^74^ Since then, an escalating trend of resistance among *A. baumannii* isolates has grown to become a concerning issue in clinical settings, ^42^ and this organism appears endemic in countries of North Africa and the Middle East, and has caused several outbreaks in European countries as well. ^75^ The documentation of CRAB was not necessarily correlated with in-hospital mortality, ^76^ but previous exposure to antibiotics and the severity of VAP were identified as risk factors for infection, and could be minimized by judicious use of carbapenems and colistin. ^77^

The rates of carbapenem resistance among *A. baumannii* in VAP and HAP is variable with some reports of 60% ^78^ and other studies with numbers as high as 86%. ^75^^,^^79^ Drug-resistant *A. baumannii* in VAP is reported to confer longer hospital stays and increased mortality, which can exceed 60%. ^80^^,^^81^ The length of hospital stay, previous antibiotic treatment, duration of mechanical ventilation, disease severity, and predominance of drug-resistant *A. baumannii* strains in hospital environments have been recognized as risk factors for VAP due to MDR *A. baumannii.* ^77^ Plasmids of CRAB isolates that harbor genetic determinants coding for different carbapenem-hydrolysing class D β-lactamases ( *bla*_OXA-23_, *bla*_OXA-58_, *bla*_OXA-58_-like, and *bla*_OXA-72_) result in high-level resistance to all carbapenems. ^42^ For example, in Brazilian university hospitals, CRAB isolates with plasmids carrying *bla*_OXA-23_ were identified in a range of about 70% ^82^ to 100% ^83^ of VAP cases. Likewise, 100% of *A. baumannii* strains identified in a study on VAP from Pakistan were CRAB, of which 95% positively amplified *bla*_OXA-23_ gene. ^84^ In China, a recent 7-year analysis of the molecular epidemiology of VAP showed that CRAB caused 65% of *A. baumannii* VAP cases, and carbapenem resistance was related to expression of OXA-23 and OXA-24, as well as efflux pump-encoding genes (AdeABC and AdeFGH). Infection with CRAB was significantly associated with longer mechanical ventilation time and longer antibiotic administration after VAP diagnosis. ^85^ Also in China, according to a retrospective analysis, CRAB-induced HAP occurred mostly in patients with underlying diseases, and in those who received antimicrobial therapies including broad-spectrum β-lactams, invasive mechanical ventilation, and catheterization. The genes encoding OXA-23 were detectable in 97% of the strains. ^86^ In Iran, as reported in an investigation of CRAB in VAP, resistance rates to both imipenem and meropenem were above 90%, and the frequencies of OXA-23 and OXA-24 were 58% and 31% respectively, with 42% of the strains harboring the insertion sequence *IS*Aba1 upstream of OXA carbapenemases capable of modulating their expression and transfer. ^87^ In Vietnam, over 80% of *A. baumannii* strains from HAP were carbapenem-resistant with the carbapenemase genes *bla*_OXA-23-like_, *bla*_OXA-58-like,_ and *bla*_NDM-1_ having rates of 78%, 10%, and 6%, respectively. ^88^ The rates of carbapenem resistance in *A. baumannii* were as high as 97% in a VAP study involving three European countries, Greece, Spain, and Italy. Resistance was associated with an acquired carbapenemase, OXA-23 (80%), OXA-40 (5%), OXA-58 (2%) or OXA-23/58 (2%). Almost 65% of CRAB isolates were XDR or pan-drug resistant, and belonged to a predominant clonal lineage, suggesting the presence of an epidemic clone and highlighting the difficulty in empirical treatment of CRAB. ^89^ The expansion of resistance in CRAB isolates and their increasing detection in nosocomial infections like HAP and VAP necessitate precise and regular programs of surveillance and control.

## Carbapenem-resistant *P. aeruginosa* (CRPA)

Although a wide spectrum of bacteria can cause HAP and VAP, *P. aeruginosa* remains one of the most frequent causative pathogens. ^41^ Multifaceted, opportunistic, and drug-resistant, *P. aeruginosa* continues to be a major source of infections in HAP and VAP with high morbidity and mortality. Its enormous potential for variation and the large number of virulence factors the pathogen has at its disposal, has allowed it to be adaptable and flexible, giving it the opportunity to customize its response to healthcare settings where it lingers as a major concern. ^90^ In 2019, the CDC reported that *P. aeruginosa* has a carbapenem resistance rate of up to 12% in the US. ^91^ An epidemiological analysis in 2015 involving 50 countries showed that the international resistance rates of *P. aeruginosa* to carbapenems vary from 10% to 60%. ^92^ Numerous resistance mechanisms drive the emergence of CRPA, most often including porin deficiency (especially OprD), efflux pump overactivity (mainly MexAB-OprM and MexCD-OprJ), and, less frequently, carbapenem-inactivating enzymes and hyperproduction of AmpC cephalosporinases. ^93^^,^^94^

In a pediatric ICU, the rate of CRPA among VAP samples was 52%, and risk factors for the infection included length of stay until the diagnosis of VAP, presence of central venous catheters, prior use of cefepime, ciprofloxacin, colistin, and teicoplanin. ^95^ Furthermore, in a study of VAP in the ICU of three hospital centers in 2020, CRPA isolates showed high resistance for both imipenem and meropenem, with respectively 74% and 68%, and were most likely to exhibit upregulation of efflux pumps or porin loss. ^61^

## Colistin-resistant Gram-negative bacteria

A cationic, polypeptide antibiotic, bactericidal against Gram-negative bacteria, colistin (polymyxin E) ^96^ has re-emerged as a possible antibiotic treatment option for MDR Gram-negative bacteria in HAP and VAP. ^97^ Its revival was prompted by its high effectiveness against MDR *A. baumannii*, *K. pneumoniae* and *P. aeruginosa.* ^98^ Polymyxins target the outer membrane of Gram-negative bacteria using electrostatic interactions that develop between the positively charged polymyxin molecule and the negatively charged phosphate group that forms part of the lipid A residue of the bacterial cell wall lipopolysaccharide (LPS). Upon binding to the LPS and phospholipids in the outer cell membrane, polymyxins competitively displace divalent cations from the phosphate groups of membrane lipids, destabilizing the outer cell membrane, and causing leakage of intracellular contents and cell death. ^99^ Undesirably, the use of polymyxins among the few enduring useable options for the therapy of MDR Gram-negative pathogens has prompted bacterial resistance. Such resistance may be chromosomal and associated with the modifications of lipid A, or may be encoded on transposable elements, namely mobile colistin resistance ( *mcr*) genes. A review of the mechanisms of Gram-negative bacterial resistance to polymyxin has been meticulously presented elsewhere. ^96^

With the increase in the magnitude of resistance towards colistin among Gram-negative bacteria, ^100^ reports of such resistance being an important drive of increased mortality are emerging. ^101^ In one survey, about 48% of *A. baumannii* isolated from patients with VAP in Greece, Italy, and Spain were colistin-resistant, with several amino acid substitutions in the PmrCAB two-component system. ^102^ With this system being responsible for addition of phosphoethanolamine to lipid A, its modifications reduce the net negative charge of the outer membrane, thereby affecting colistin binding and preventing loss of integrity and disruption of the cell membrane. ^103^ Likewise, in 2015, colistin-resistant *A. baumannii* isolates were identified at a hospital system in Pennsylvania from patients with VAP, with lipid A modification by the addition of phosphoethanolamine accounting for colistin resistance. ^104^

A prospective cohort research done in 2022 on MDR Gram-negative pathogens recovered from intubated patients with VAP showed colistin resistance rates of 3-20% among MDR *A. baumannii*, *K. pneumoniae*, as well as *P. aeruginosa.* ^105^ Also, in a 5-year retrospective study following over 5,500 patients in four general ICUs in Barcelona, Spain, the rate of colistin resistance among *P. aeruginosa* isolated from HAP or VAP specimens was 15%. ^106^ These numbers warrant careful attention to colistin use, and thorough re-evaluation of its introduction to antimicrobial therapy. According to an expert opinion published in 2021, the use of colistin in VAP has limited efficacy and significant nephrotoxicity. ^107^ The addition of the drug to the medication regimen did not show significant differences in patient mortality, ^105^ indicating that its use should rather be reassessed as newer agents become available, to prevent further buildup of colistin resistance among MDR Gram-negative pathogens.

## MDR Gram-negative pathogens causing pneumonia in COVID-19 patients

The coronavirus disease 2019 (COVID-19) caused by the severe acute respiratory syndrome coronavirus 2 (SARS-CoV-2) has made a serious public health threat worldwide with different populations at risk. ^108^ The pandemic has resulted in millions of infections globally and has stressed both the healthcare and the economic systems to the extreme, ^109^ with over 650 million cases confirmed and a death toll reaching almost 7 million bereavements worldwide, as of November 2022. ^110^ Severe COVID-19 has sculpted critical challenges for research and medical communities, with older age, male sex, and comorbidities (hypertension, diabetes, cardiovascular disease, chronic pulmonary disease, chronic kidney disease, chronic liver disease, and cancer) being the common risks for severe disease. Given that SARS-CoV-2 viral entry is primarily through the respiratory tract, upper and lower respiratory tract involvement is the most common manifestation. ^111^ Observational studies report that COVID-19 patients suffer from secondary bacterial infections, worsening the disease and increasing mortality, particularly in those who require invasive mechanical ventilation. However, the rates of these bacterial co-infections and secondary infections remained low (5-7%), ^112^^,^^113^ except for critically sick ICU patients who had higher rates. ^114^ Numerous studies of COVID-19 patients admitted to the ICU show that most patients were empirically administered antibiotics, increasing the prevalence of MDR pathogens. ^114^^,^^115^ Prevalence of MDR Gram-negative bacteria is also attributed to improper compliance of hand hygiene and contact isolation, such as glove and gown usage, by hospital staff. ^116^ Moreover, given that such errors also increase HAP and VAP occurrence among COVID-19 patients, specific infection prevention strategies (such as preventing reintubation, minimizing sedation, providing early enteral nutrition, among others) have become increasingly pivotal to implement. ^117^ In general, the rates of VAP in patients with COVID-19 who were critically ill was reported to rise above 50%, ^118^^–^^122^ with an estimated mortality exceeding 40%, and an increase in the number of patients requiring intensive care. ^123^ In a multicenter study, VAP accounted for 50% of all hospital-acquired infections in patients with COVID-19, with 28% prevalence of Gram-negative pathogens as agents in VAP. ^124^ In another case series, 2% of HAP and 33% of VAP were documented among COVID-19 patients admitted to the ICU, with VAP resulting in more acute respiratory distress syndrome (ARDS), and being associated with more acute kidney injury, longer mechanical ventilation, and longer ICU stay. ^125^

Across studies of COVID-19 patients diagnosed with VAP, frequent growth of Gram-negative bacteria has been revealed in multiple studies, with predominantly high rates of *P. aeruginosa.* ^123^ In 2022, Velásquez-Garcia and Colleagues ^126^ conducted a systematic review on causative agents of VAP and their antibiotic resistance patterns in COVID-19 patients and most collected studies were from France (32%), Italy (20%), Spain (12%), as well as the United States (8%). The prevalence of Gram-negative bacteria was highest in VAP, with ranges of 7.5-72.5% for *P. aeruginosa*, 6.9-43.7% for *K. pneumonia*, 1.6-20% for *E. cloacae*, and 1.2-20% for *A. baumannii.* Likewise, a late systematic literature review summarizing available evidence regarding VAP in patients undergoing mechanical ventilation because of ARDS secondary to SARS-CoV-2 infection, reported Gram-negative bacteria to be the predominant microorganisms (>70% in most series) followed by Gram-positive bacteria (mostly *S. aureus*). Also, since most cases of COVID-19-related VAP are diagnosed more than 7 days from initiation of invasive mechanical ventilation, patients are at increased risk for MDR strains. ^127^

Only few studies investigated the susceptibility patterns among Gram-negative pathogens, and the main resistance mechanisms reported by these studies include the production by Gram-negative pathogens of ESBL, AmpC, and carbapenemases. Table 1 summarizes some findings of Gram-negative pathogens described in this review in patients with COVID-19-related VAP and remarkable data about their resistance. The published research revealed only limited information on the patterns of antibiotic resistance, as well as scarce data from low- and middle-income countries. As such, every effort should be implemented for monitoring and preventing these infections in the light of COVID-19 and bacterial resistance, and for improving health systems’ preparedness for future pandemics.

**Table 1.** Examples of VAP studies from COVID-19 patients and related Gram-negative pathogens, with a summary of resistance findings.

Country	Study setting/design	Gram-negative pathogens	Associated resistance type or mechanism	Reference
Italy	Multicenter, ICU	35% *Pseudomonas aeruginosa* 19% *Klebsiella pneumoniae*	32% of Gram-negative pathogens were carbapenem-resistant	^128^
Italy	Retrospective, observational in two ICUs of one hospital	*Pseudomonas* spp. accounted for 83% of VAP cases	84% of *Pseudomonas* spp. were carbapenem-resistant One out of 19 patients had co-infection with colistin resistant *Serratia marcescens*	^129^
France	Retrospective, single-center in the ICU	Incidence of VAP due to MDR was significantly higher in COVID-19 patients (48%) versus non-COVID-19 patients (16%)	18% of VAP cases were caused by ESBL-producing *Enterobacterales*, and 2% by CRE (including OXA-48 and NDM producing organisms)	^62^
France	Retrospective cohort study in a single ICU	*Enterobacterales* accounted for 70% of VAP cases *Pseudomonas aeruginosa* accounted for 37% of VAP cases	40% of *Enterobacterales* were AmpC-cephalosporinase producers 6% of *Enterobacterales* were AmpC-cephalosporinase producers	^130^
United States	Observational, single-center in the ICU	33% of VAP episodes were caused by MDR organisms including primarily *P. aeruginosa* and *Enterobacterales*	Not specified	^131^
Switzerland	Cohort, single-center, prospective study among ICU patients	*P. aeruginosa* (46%) and *Enterobacterales* (36%) comprised the two largest etiologic groups	50% of *P. aeruginosa* were carbapenem resistant	^132^
Spain	Retrospective, single center in the ICU	*P. aeruginosa* comprised 38% of the cases of VAP, and it was the third most frequent resistant organism after *S. aureus* and *Enterococcus faecium*	Not specified	^133^
Belgium	Retrospective, single-center in the ICU	44% *Klebsiella* spp. 18% *P. aeruginosa* 11% *Enterobacter* spp.	29% of agents causing VAP were ESBL-producing *Klebsiella* spp., and 5% were extensively drug-resistant *P. aeruginosa* producing VIM which confers resistance to all tested antibiotics except aztreonam	^134^
Egypt	Two university hospitals with COVID-19 patient admission	Gram-negative isolates were predominant (above 70%) 28.5% *K. pneumoniae* 16.6% *A. baumannii* 9.5% *Escherichia coli* 9.5% *P. aeruginosa* 4.7% *Enterobacter cloacae* Fungal infections, caused by *Candida* (12%), were all mixed with bacteria	NDM-1 was the most predominant antibiotic resistance gene (55%), followed by CTX-M (52%), then TEM (41%), KPC (34%) and SHV (7%)	^135^

VAP=Ventilator-Associated Pneumonia; ICU=Intensive Care Unit; MDR=Multidrug-resistant; ESBL=Extended-Spectrum β-Lactamase; CRE=Carbapenem-Resistant *Enterobacterales*.

## Conclusions

With the above data, it is evident that the advent of Gram-negative pathogens with important antimicrobial resistance profiles has mounted with time in HAP and VAP, hindering treatment and adversely affecting clinical outcomes. Moreover, the increased recognition of these pathogens in patients with COVID-19 backs up recent distresses regarding the emergence of MDR bacterial co-infections during the global pandemic. Such public health issues necessitate more stringent implementation of hand hygiene, contact isolation and other VAP prevention strategies such as preventing reintubation and minimizing sedation for patients. As such, the development of approaches for alleviating the effect of these infections in this patient population should be a compulsory element of management tactics for COVID-19 patients, and part of the alertness for future outbreaks. It is evident that the dwindling antibiotic pipeline, and the major decline in the approval of new antibiotics or new classes, are exerting precarious pressure on demanding infections like HAP and VAP. As such, more research, more funding, and more focus into advanced surveillance methods like whole genome sequencing, and innovative methods of antibiotic discovery like deep learning and artificial intelligence to address MDR Gram-negative pathogens, will ultimately influence the presentation of HAP and VAP, as well as other threatening infections.

## Data Availability

No data are associated with this article.
