# Supplementary material for: Hospital-acquired and ventilator-associated pneumonia caused by multidrug-resistant Gram-negative pathogens: Understanding epidemiology, resistance patterns, and implications with COVID-19
Source: F1000Res. 2024 Mar 25;12:92. Originally published 2023 Jan 24. [Version 2] doi: 10.12688/f1000research.129080.2 (PMC11195619; doi:10.12688/f1000research.129080.2)
Supplement: Peer review history

# Reviewer report: Reviewer response for version 2

**DOI:** 10.5256/f1000research.163885.r258906

**Published:** 2024-06-18

**Authors:** Ghassan Dbaibo

**Affiliations:** 1 Center for Infectious Diseases Research, Division of Pediatric Infectious Diseases, Department of Pediatrics and Adolescent Medicine, American University of Beirut Medical Center, Beirut, Lebanon

I have reviewed the responses and now find the paper acceptable for indexing.

Is the review written in accessible language?

Partly

Are all factual statements correct and adequately supported by citations?

Partly

Are the conclusions drawn appropriate in the context of the current research literature?

Yes

Is the topic of the review discussed comprehensively in the context of the current literature?

Yes

Reviewer Expertise:

Infectious diseases and sphingolipid biochemistry

I confirm that I have read this submission and believe that I have an appropriate level of expertise to confirm that it is of an acceptable scientific standard.

# Reviewer report: Reviewer response for version 2

**DOI:** 10.5256/f1000research.163885.r258905

**Published:** 2024-04-17

**Authors:** Arjana Tambić Andrašević

**Affiliations:** 1 University Hospital for Infectious Disease Fran Mihaljevic, Zagreb, Croatia; 2 Department of Clinical Microbiology, School of Dental Medicine, University of Zagreb, Zagreb, City of Zagreb, Croatia

I have no new comments

Is the review written in accessible language?

Yes

Are all factual statements correct and adequately supported by citations?

Yes

Are the conclusions drawn appropriate in the context of the current research literature?

Yes

Is the topic of the review discussed comprehensively in the context of the current literature?

Partly

Reviewer Expertise:

Antibiotic resistance surveilalnce

I confirm that I have read this submission and believe that I have an appropriate level of expertise to confirm that it is of an acceptable scientific standard.

# Reviewer report: Reviewer response for version 1

**DOI:** 10.5256/f1000research.141737.r195137

**Published:** 2023-10-09

**Authors:** Arjana Tambić Andrašević

**Affiliations:** 1 University Hospital for Infectious Disease Fran Mihaljevic, Zagreb, Croatia; 2 Department of Clinical Microbiology, School of Dental Medicine, University of Zagreb, Zagreb, City of Zagreb, Croatia

The article describes the epidemiology, risk factors and aetiology of hospital-acquired pneumonia (HAP) and ventilator-associated pneumonia (VAP) indicating that multidrug resistant (MDR) gram-negative bacteria are on the rise. Incidence of MDR bacteria differs from country to country and authors are citing many studies on incidence of these pathogens in VAP and in intensive care units from different parts of the world. Authors are giving a nice review of resistance mechanisms to critically important antibiotics, colistin and beta-lactams, carbapenems in particular. Special attention is given to the high incidence of MDR pathogens causing VAP in COVID patients. Importance of close monitoring of VAP and HAP aetiology is stressed as well as the importance of investing in development of new antibiotics. However, more should be discussed on the role of infection prevention and control in reducing VAP and HAP and in particular infections caused by MDR bacteria.

 Specific comments:

1. *Numerous resistance mechanisms drive the emergence of CRPA, most often including porin deficiency (especially OprD), efflux pump overactivity (mainly MexAB-OprM and MexCD-OprJ), and, less frequently, carbapenem-inactivating enzymes.* – add important mechanism: hyperproduction of AmpC cephalosporinases
2. *Numerous studies of COVID-19 patients admitted to the ICU show that most patients were empirically administered antibiotics, increasing the prevalence of MDR pathogens.111,112 *

The review should also reflect on the following: Incidence of VAP caused by MDRO was increased in COVID patients also due to the poor implementation of hand hygiene and standard precautions and improper use of gloves in cohorted COVID patients. In general, the role of infection prevention and control in reducing VAP and HAP and MDR spread should be more discussed.

  

1. *Conclusions*: the importance of improving hand hygiene, standard precautions and contact isolation in prevention of MDRO spread and specific measures to prevent VAP should be mentioned in conclusions

Is the review written in accessible language?

Yes

Are all factual statements correct and adequately supported by citations?

Yes

Are the conclusions drawn appropriate in the context of the current research literature?

Yes

Is the topic of the review discussed comprehensively in the context of the current literature?

Partly

Reviewer Expertise:

Antibiotic resistance surveilalnce

I confirm that I have read this submission and believe that I have an appropriate level of expertise to confirm that it is of an acceptable scientific standard.

# Response

**Published:** 2024-03-15

**Authors:** Dalal Hammoudi Halat

**Affiliations:** Lebanese International University, Lebanon

The article describes the epidemiology, risk factors and aetiology of hospital-acquired pneumonia (HAP) and ventilator-associated pneumonia (VAP) indicating that multidrug resistant (MDR) gram-negative bacteria are on the rise. Incidence of MDR bacteria differs from country to country and authors are citing many studies on incidence of these pathogens in VAP and in intensive care units from different parts of the world. Authors are giving a nice review of resistance mechanisms to critically important antibiotics, colistin and beta-lactams, carbapenems in particular. Special attention is given to the high incidence of MDR pathogens causing VAP in COVID patients. Importance of close monitoring of VAP and HAP aetiology is stressed as well as the importance of investing in development of new antibiotics. However, more should be discussed on the role of infection prevention and control in reducing VAP and HAP and in particular infections caused by MDR bacteria.

**We thank the reviewer for their opinion on the article, and we will address below the comments suggested to improve the contents. Our point-by-point itemized responses are shown below each comment in bold face.**

 Specific comments:

1. *Numerous resistance mechanisms drive the emergence of CRPA, most often including porin deficiency (especially OprD), efflux pump overactivity (mainly MexAB-OprM and MexCD-OprJ), and, less frequently, carbapenem-inactivating enzymes.* – add important mechanism: hyperproduction of AmpC cephalosporinases 
**We agree with the reviewer and the above mechanism has now been added in the manuscript in page 8, in 2 ^nd^ paragraph, with the below reference:**
** Rodríguez-Martínez JM, Poirel L, Nordmann P. Extended-spectrum cephalosporinases in Pseudomonas aeruginosa. *Antimicrob Agents Chemother.* 2009;53(5):1766-1771. doi:10.1128/AAC.01410-08**

**The editorial team will kindly adjust the numbering of references in the manuscript.**

  

1. *Numerous studies of COVID-19 patients admitted to the ICU show that most patients were empirically administered antibiotics, increasing the prevalence of MDR pathogens.111,112*

The review should also reflect on the following: Incidence of VAP caused by MDRO was increased in COVID patients also due to the poor implementation of hand hygiene and standard precautions and improper use of gloves in cohorted COVID patients. In general, the role of infection prevention and control in reducing VAP and HAP and MDR spread should be more discussed.

**We have added the following lines and listed two references in page 11, 1 ^st^ paragraph, line 8:**

**“Prevalence of MDR Gram-negative bacteria is also attributed to improper compliance of hand hygiene and contact isolation, such as glove and gown usage, by hospital staff. (Mills & Marchaim, 2021). Moreover, given that such errors also increase HAP and VAP occurrence among COVID-19 patients, specific infection prevention strategies (such as preventing reintubation, minimizing sedation, providing early enteral nutrition, among others) have become increasingly pivotal to implement. (Klompas , et al. 2022)”**

1. **Mills JP, Marchaim D. Multidrug-Resistant Gram-Negative Bacteria: Infection Prevention and Control Update. *Infect Dis Clin North Am.* 2021;35(4):969-994. doi:10.1016/j.idc.2021.08.001**
2. **Klompas M, Branson R, Cawcutt K, et al. Strategies to prevent ventilator-associated pneumonia, ventilator-associated events, and nonventilator hospital-acquired pneumonia in acute-care hospitals: 2022 Update. *Infect Control Hosp Epidemiol.* 2022;43(6):687-713. doi:10.1017/ice.2022.88**

**The editorial team will kindly adjust the numbering of references in the manuscript.**

*Conclusions*: the importance of improving hand hygiene, standard precautions and contact isolation in prevention of MDRO spread and specific measures to prevent VAP should be mentioned in conclusions

**We agree with the reviewer, and have added the following lines to page 13, in the Conclusion section:**

**“Such public health issues necessitate more stringent implementation of hand hygiene, contact isolation and other VAP prevention strategies such as preventing reintubation and minimizing sedation for patients.**”

# Reviewer report: Reviewer response for version 1

**DOI:** 10.5256/f1000research.141737.r163960

**Published:** 2023-04-24

**Authors:** Ghassan Dbaibo

**Affiliations:** 1 Center for Infectious Diseases Research, Division of Pediatric Infectious Diseases, Department of Pediatrics and Adolescent Medicine, American University of Beirut Medical Center, Beirut, Lebanon

This is an excellent current review of the topic of hospital-acquired pneumonia and ventilator-associated pneumonia in the setting of increasing resistance among the responsible Gram-negative organisms.

1. There are some missing references.
2. There are some problems with grammar, spelling, and syntax that could be improved
3. Title maybe a bit misleading “Bacterial Pneumonia” since the focus is on HAP and VAP.
4. Methodology is absent. Perhaps a statement indicating how the authors decided to perform this review would be appropriate. How did they choose the references in this review, including some but not others? What questions were they looking to answer? If this is a descriptive review, what is the scope (geography, age, setting, time-frame, etc.)?
5. It may have made more sense to start with ESBL Enterobacterales since they emerged first, among the categories highlighted, followed by the others (instead of starting with carbapenem-resistant organisms).
6. Page 4, third paragraph (of the pdf version of the article): “Evidence indicates….”, this statement requires at least one, preferably more references.
7. Page 4, third paragraph: “Pneumonia etiology is thought…”, this should be HAP etiology, as overall pneumonia is still dominated by *S. pneumoniae*.
8. Page 4, last paragraph: “Parallel results were reported…”, do the authors mean “Similar results were….”?
9. Throughout: full bacterial name only needed with first mention, subsequently abbreviation can be used, e.g. *S. maltophilia* (misspelled on at least one occasion)
10. 1Page 6, first paragraph: parentheses not closed ( *bla…*)
11. Throughout: The resistance enzymes should be presented consistently, e.g. *bla*- _OXA_ versus OXA
12. Page 6, second paragraph, line 7: “wide range” seems to be misplaced between the lower and upper limit
13. Page 6, third paragraph, line 3: “opposed” should be replaced by “compared”, it reads better

Is the review written in accessible language?

Partly

Are all factual statements correct and adequately supported by citations?

Partly

Are the conclusions drawn appropriate in the context of the current research literature?

Yes

Is the topic of the review discussed comprehensively in the context of the current literature?

Yes

Reviewer Expertise:

Infectious diseases and sphingolipid biochemistry

I confirm that I have read this submission and believe that I have an appropriate level of expertise to confirm that it is of an acceptable scientific standard, however I have significant reservations, as outlined above.

# Response

**Published:** 2024-03-15

**Authors:** Dalal Hammoudi Halat

**Affiliations:** Lebanese International University, Lebanon

This is an excellent current review of the topic of hospital-acquired pneumonia and ventilator-associated pneumonia in the setting of increasing resistance among the responsible Gram-negative organisms.

**We thank the reviewer for their opinion on the article, and we will address below their comments to improve the contents. Our point-by-point itemized responses are shown below each comment in bold face.**

1. There are some missing references.
**Upon recommendation of the reviewer, we have added a total of five new references, in pages 4, 8 and 11 of the article. They are as follows:**
  1. **Gadsby NJ, Musher DM. The Microbial Etiology of Community-Acquired Pneumonia in Adults: from Classical Bacteriology to Host Transcriptional Signatures. *Clin Microbiol Rev.* 2022;35(4):e0001522. doi:10.1128/cmr.00015-22**
  2. **Miyashita N. Atypical pneumonia: Pathophysiology, diagnosis, and treatment. *Respir Investig.* 2022;60(1):56-67. doi:10.1016/j.resinv.2021.09.009**
  3. **Rodríguez-Martínez JM, Poirel L, Nordmann P. Extended-spectrum cephalosporinases in Pseudomonas aeruginosa. *Antimicrob Agents Chemother.* 2009;53(5):1766-1771. doi:10.1128/AAC.01410-08**
  4. **Mills JP, Marchaim D. Multidrug-Resistant Gram-Negative Bacteria: Infection Prevention and Control Update. *Infect Dis Clin North Am.* 2021;35(4):969-994. doi:10.1016/j.idc.2021.08.001**
  5. **Klompas M, Branson R, Cawcutt K, et al. Strategies to prevent ventilator-associated pneumonia, ventilator-associated events, and nonventilator hospital-acquired pneumonia in acute-care hospitals: 2022 Update. *Infect Control Hosp Epidemiol.* 2022;43(6):687-713. doi:10.1017/ice.2022.88**

**The editorial team will kindly** **adjust the numbering of references in the manuscript.**

  

1. There are some problems with grammar, spelling, and syntax that could be improved
   
**As advised by the reviewer,** **we have improved the above aspects of the article.**
2. Title maybe a bit misleading “Bacterial Pneumonia” since the focus is on HAP and VAP.
**Upon recommendation of the reviewer, the title was modified to indicate focus on HAP and VAP.** 
3. Methodology is absent. Perhaps a statement indicating how the authors decided to perform this review would be appropriate. How did they choose the references in this review, including some but not others? What questions were they looking to answer? If this is a descriptive review, what is the scope (geography, age, setting, time-frame, etc.)?
**We thank the reviewer for this question. Since this was a narrative review, a statement indicating how the search was done was added at the end of the introduction section.**
4. It may have made more sense to start with ESBL Enterobacterales since they emerged first, among the categories highlighted, followed by the others (instead of starting with carbapenem-resistant organisms).

**We agree with the reviewer, and we have** **reorganized the paragraphs according to the request. We presented the organisms with the following order in the manuscript: extended-spectrum β-lactamase (ESBL)-producing *Enterobacterales,* carbapenem-resistant *Enterobacterales* (CRE), carbapenem-resistant *Acinetobacter baumannii* (CRAB), carbapenem-resistant *P. aeruginosa* (CRPA), and colistin-resistant Gram-negative pathogens.**

**Kindly adjust the numbering of references in the manuscript.**

1. Page 4, third paragraph (of the pdf version of the article): “Evidence indicates….”, this statement requires at least one, preferably more references.

**We agree with the reviewer, and we have added the following two references, in page 4, at the end of the mentioned paragraph:**

1.
  1. **Gadsby NJ, Musher DM. The Microbial Etiology of Community-Acquired Pneumonia in Adults: from Classical Bacteriology to Host Transcriptional Signatures. *Clin Microbiol Rev.* 2022;35(4):e0001522. doi:10.1128/cmr.00015-22**
  2. **Miyashita N. Atypical pneumonia: Pathophysiology, diagnosis, and treatment. *Respir Investig.* 2022;60(1):56-67. doi:10.1016/j.resinv.2021.09.009**

 

**The editorial team will kindly adjust the numbering of references in the manuscript.**

  

1. Page 4, third paragraph: “Pneumonia etiology is thought…”, this should be HAP etiology, as overall pneumonia is still dominated by *S. pneumoniae*.
**Indeed, we agree on this comment from the reviewer, and this was stated at the beginning of this section. The sentence mentioned has been adjusted accordingly.**
2. Page 4, last paragraph: “Parallel results were reported…”, do the authors mean “Similar results were….”?
** The sentence was adjusted accordingly.**
3. Throughout: full bacterial name only needed with first mention, subsequently abbreviation can be used, e.g. *S. maltophilia* (misspelled on at least one occasion)
**We have adjusted the bacterial names, as advised, and corrected the spelling errors.**
4. Page 6, first paragraph: parentheses not closed ( *bla…*)
**As advised by the reviewer, we have closed the parentheses in above mentioned line.**
5. Throughout: The resistance enzymes should be presented consistently, e.g. *bla*- _OXA_ versus OXA.

**We thank the reviewer for thorough review of the writing. The manuscript was checked to ensure all enzymes are represented by name (example: OXA), while reference to genes included *bla* (example: *bla*- _OXA_ ).**


**   **

1. Page 6, second paragraph, line 7: “wide range” seems to be misplaced between the lower and upper limit
**We have removed, as advised, the terms “wide range” from the mentioned line.**
2. Page 6, third paragraph, line 3: “opposed” should be replaced by “compared”, it reads better
**We have replaced, as advised, the mentioned term with “compared”.**
